# Frequency and phenotype of natural killer cells and natural killer cell subsets in bovine lymphoid compartments and blood

**DOI:** 10.1111/imm.12708

**Published:** 2017-02-07

**Authors:** Carly A. Hamilton, Suman Mahan, Charlotte R. Bell, Bernardo Villarreal‐Ramos, Bryan Charleston, Gary Entrican, Jayne C. Hope

**Affiliations:** ^1^ The Roslin InstituteUniversity of EdinburghMidlothianUK; ^2^ ZoetisKalamazooMIUSA; ^3^ Animal and Plant Health AgencyWeybridge, AddlestoneUK; ^4^ The Pirbright InstituteWokingSurreyUK; ^5^ Moredun Research InstitutePentlands Science ParkMidlothianUK

**Keywords:** natural killer cells, recirculation

## Abstract

Natural killer (NK) cells are widely distributed in lymphoid and non‐lymphoid tissues, but little is known about the recirculation of NK cells between blood and tissues. This is relevant to understanding recirculation in the steady‐state and also for determining the roles for NK cells in vaccine‐induced immunity and responses to infection. Therefore, the percentage of NK cells and their phenotype across peripheral blood, afferent lymph and lymph nodes in steady‐state conditions was investigated in cattle using the pseudo‐afferent lymphatic cannulation model. CD2^+^ CD25^lo^ NK cells were the predominant subset of NK cells within the blood. In contrast, CD2^−^ CD25^hi^ NK cells were the main subset present within the skin‐draining afferent lymphatic vessels and lymph nodes, indicating that CD2^−^ NK cells are the principal NK cell subset trafficking to lymph nodes via the afferent lymphatic vessel. Furthermore, a low percentage of NK cells were present in efferent lymph, which were predominantly of the CD2^−^ subset, indicating that NK cells can egress from lymph nodes and return to circulation in steady‐state conditions. These compartmentalization data indicate that NK cells represent a population of recirculating lymphocytes in steady‐state conditions and therefore may be important during immune responses to vaccination or infection.

**Abbreviations**
AL: afferent lymph
APHA: Animal and Plant Health Agency
EL: efferent lymph
LN: lymph node
NK: natural Killer
PB: peripheral blood
S1P1: sphingosine‐phosphate‐receptor 1
S1P5: sphingosine‐phosphate‐receptor 5

## Introduction

Natural killer (NK) cells are large granular lymphocytes that were first identified in the 1970s from their ability to lyse malignant or transformed cells without previous sensitization.^1^ This heterogeneous cell population has diverse roles in the immune system and is the first line of defence in the control of viruses, bacteria and parasites.^2^, ^3^, ^4^, ^5^ NK cells are widely distributed in lymphoid and non‐lymphoid compartments,^6^, ^7^, ^8^, ^9^, ^10^ allowing them to respond quickly to infection or inflammation through cytotoxicity and production of cytokines. Defining the precise anatomical location and phenotypic characteristics of NK cells in steady‐state conditions serves as a benchmark for studying their roles in inflammation and responses to vaccines and pathogens. Furthermore, NK cells can interact with innate accessory cell populations including dendritic cells to drive adaptive immune responses. Identifying the location of these interactions *in vivo* would provide opportunities for specific targeting to improve vaccine delivery.

Lymphocytes continuously circulate between the blood and tissues such as the skin via the lymphatics and lymph nodes (LNs) in steady‐state conditions. This continuous recirculation is required for immune surveillance.^11^ Lymphocytes can enter LNs by two routes, first from the circulation through high endothelial venules and second, they can migrate from the tissues through afferent lymphatic vessels. Lymphocytes can then egress from the LNs through the efferent lymphatic vessel and return to the circulation via the thoracic duct.^12^ In contrast to the re‐circulatory nature of *αβ* T cells, very little is known about NK cell recirculation.^13^ It has been demonstrated in mice that NK cells migrate from the circulation to LNs via high endothelial venules.^14^, ^15^ NK cells are present in human afferent lymphatic vessels^16^, ^17^, ^18^ and one study has shown that NK cells are found in human efferent lymph (EL).^19^

In cattle, NK cells are defined by their constitutive expression of the natural cytotoxicity receptor, NKp46 (CD335)^20^ and, similar to human^21^ and murine^22^ NK cells, bovine NK cells exist as distinct subsets with bovine NKp46^+^ NK cells being subdivided into two subsets based on their differential expression of CD2.^20^ CD2^+^ NK cells are the principal subset of NK cells found in bovine peripheral blood (PB) whereas CD2low or CD2negative (referred to as CD2^−^ herein) NK cells are the main subset present in the LNs. It was demonstrated recently that bovine NK cells are present in the skin‐draining afferent lymphatic vessels of cattle in steady‐state conditions. In that study, the frequency, phenotype and function of NK cells from PB and afferent lymph (AL) of different individual calves were compared.^23^ Data presented herein aimed to extend these findings by analysing the frequency and phenotype of NK cells and NK cell subsets in parallel from the PB, AL and LNs of the same animal(s), by using the bovine pseudo‐afferent lymphatic cannulation model.^24^ This technique is well established in cattle, sheep, pigs and rats^24^, ^25^, ^26^, ^27^ and is an excellent model to study innate immune responses during vaccination or infection. In addition, we further extend these data to efferent lymph and skin.

## Materials and methods

#### Experimental animals

Experiments were performed using male British Holstein‐Friesian (*Bos taurus*) calves. Pseudo‐afferent lymphatic cannulation was performed on calves housed at the University of Edinburgh's farm according to Home Office guidelines and with approval from The Roslin Institute Local Ethics Committee. Cannulation of efferent lymphatic vessels was performed on calves that were housed at Animal and Plant Health Agency (APHA) with approval from APHA Animal Use Ethics Committee. All calves were cannulated at between 3 and 6 months of age.

#### Pseudo‐afferent lymphatic cannulation

Surgical cannulation of skin‐draining afferent lymphatic vessels was performed in seven calves at 3–6 months old, according to a protocol described in detail elsewhere.^24^ Briefly, prescapular LNs were excised under general anaesthesia and the calves were allowed to recover. LNs were collected into ice‐cold PBS, processed to release cells and mononuclear cells were obtained by density gradient centrifugation using Histopaque 1083 (Sigma Aldrich, Dorset, UK). After 8 weeks, following re‐anastomosis of the small afferent lymphatic vessels to the larger efferent lymphatic vessel, a sterile cannula was inserted into the ‘pseudo‐afferent’ lymphatic vessel. Lymph was collected into a tissue‐culture flask, secured to the side of the calf's head using a head‐collar and containing 3 g of sodium benzylpenicillin (Schering‐Plough, Kenilworth, NJ) dissolved in 5000 i.u/ml heparin (GP Pharmaceuticals, Barcelona, Spain). Flasks were changed twice daily, or as required, for up to 28 days post‐cannulation. Whole lymph was centrifuged to pellet the cells. Samples of PB were taken during the cannulation period by jugular venepuncture using 10‐ml vacutainers containing sodium heparin (10 U/ml), and peripheral blood mononuclear cells were subsequently separated by density gradient centrifugation using Histopaque 1083 (Sigma Aldrich).

#### Efferent lymphatic cannulation

Efferent lymphatic vessels of five male British Holstein‐Friesian (*B. taurus*) calves aged 3–6 months old were surgically cannulated at APHA using a method described previously.^28^ Samples of PB were taken during the cannulation period by jugular venepuncture into 10‐ml vacutainers containing sodium heparin (10 U/ml).

#### Isolation of mononuclear cells from the skin

Small sections of shaved skin were digested in RPMI‐1640 medium containing dispase I (Roche, Burgess Hill, UK; 20 μg/ml) and collagenase (Sigma Aldrich; 75 U/ml) for 90 min at 37° in a shaking incubator. The digest was then homogenized and filtered through a 40‐μm cell strainer to obtain mononuclear cells.

#### Multi‐colour immunofluoresence staining

NK cells were identified by labelling cells with mouse anti‐bovine CD3 (MM1A, IgG1, VMRD, WA) indirectly conjugated to goat anti‐mouse IgG1‐AF647 (Life Technologies, Warrington, UK), phycoerythrin‐conjugated mouse anti‐bovine CD335 (AKS1, IgG1; Bio‐Rad AbD Serotec, Kidlington, UK) or mouse anti‐bovine CD335 (AKS6, IgG2b; a gift from Professor A.K. Storset) indirectly conjugated to goat anti‐mouse IgG2b‐FITC (Southern Biotech, Birmingham, AL). Subsets of NK cells were differentiated by labelling with mouse anti‐bovine CD2‐FITC (CC42, IgG1; Bio‐Rad AbD Serotec) or mouse anti‐bovine CD2 (IL‐A42; The Pirbright Institute, Woking, UK) and goat anti‐mouse IgG2a‐AF647 (Life Technologies). Expression of CD25 was assessed by labelling NK cells with mouse anti‐bovine CD25 (CACT108A, IgG2a; VMRD, Pullman, WA) and goat anti‐mouse IgG2a‐AF647 (Life Technologies). Expression of CD62L was assessed by labelling NK cells with CD62L‐FITC (CC32, IgG1; Bio‐Rad AbD Serotec). Flow cytometry was performed on a BD LSR Fortessa with facs diva software (BD Biosciences, Oxford, UK). Gates were established by using Fluorescence Minus One controls and data were analysed using flowjo v10 software (Treestar Inc., Ashland, OR ).

#### Data analysis

Data analysis was performed using microsoft excel 2010 and graphpad prism 6 (GraphPad, San Diego, CA). Statistical analysis was completed using minitab v16 (Minitab Ltd, Coventry, UK). Distribution of data was assessed using a normality test with *P* > 0·05 deemed to be normally distributed data. Statistical methods used are detailed in individual figure legends.

## Results

### CD2^−^ NK cells are the predominant subset of NK cells present within skin‐draining AL

**Figure 1:**
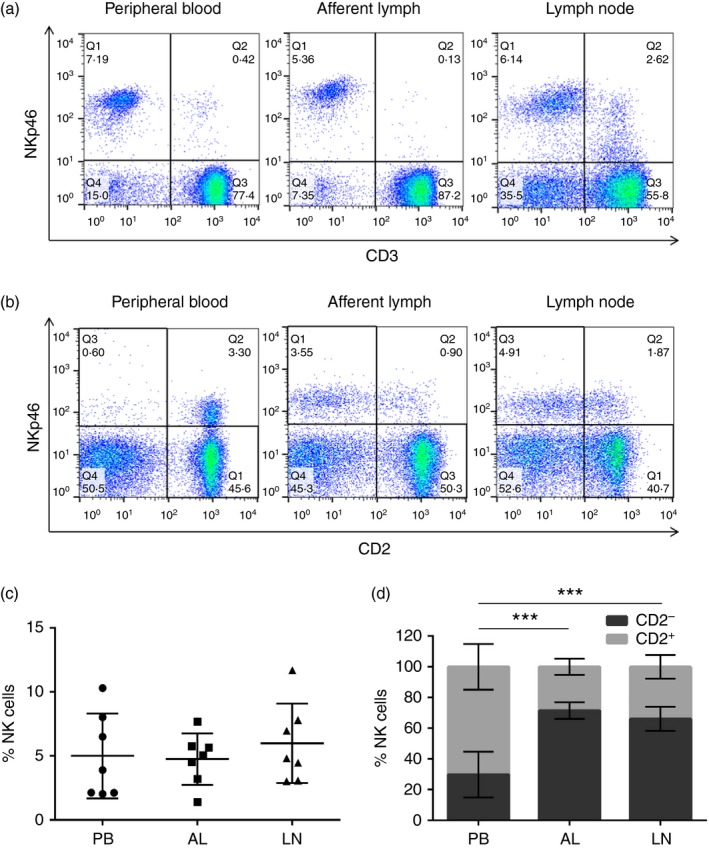
CD2^−^ natural killer (NK) cells are the predominant subset of NK cells present within skin‐draining afferent lymph (AL). Lymphocytes derived from peripheral blood (PB), AL and the lymph nodes (LNs) of seven calves were labelled with monoclonal antibodies to NKp46, CD3 and CD2 and analysed by flow cytometry. FACS plots from one representative animal illustrate the expression of NKp46 and CD3 by PB‐, AL‐ and LN‐derived lymphocytes (a). FACS plots from one representative animal illustrate the expression of NKp46 and CD2 by PB‐, AL‐ and LN‐derived lymphocytes (b). Gates were set based on Fluorescence Minus One controls. Pooled data from seven calves indicate the average percentage of NKp46^+^ CD3^−^ NK cells ± SD present in PB (circles), AL (squares) and LNs (triangles) (c). Pooled data from seven calves illustrate the average percentage of CD2^+^ (lighter bars) and CD2^−^ (darker bars) NK cells ± SD within the total gated NKp46^+^ NK cell population from PB, AL or the LNs (d). Data were normally distributed (*P* > 0·05). Significance between PB and AL was assessed using a paired *t*‐test and significance between PB and LN was assessed using a two‐sample *t*‐test. *P* < 0·001***. [Colour figure can be viewed at wileyonlinelibrary.com]

First, to determine if NK cells were present in skin‐draining afferent lymphatic vessels and to compare their frequency to those present in PB and LNs, the abundance of NKp46^+^ CD3^−^ NK cells within PB, AL and LNs of seven calves was investigated. The gating strategy used to identify lymphocytes throughout this study is illustrated in the Supplementary material (Fig. S1). NKp46^+^ CD3^−^ NK cells represented 5·1% (2·05–10·3%; SD = 3·1), 4·8% (1·39–7·68%; SD = 1·9) and 6% (3·03–11·7%; SD = 2·9) of lymphocytes in PB, AL and the LNs, respectively (representative dot plots are shown in Fig. 1a). No significant differences were evident between the percentages of NK cells present across the three compartments (Fig. 1c). CD2^+^ NK cells were the predominant subset in PB, but CD2^−^ NK cells were the principal NK cell subset found in the skin‐draining afferent lymphatic vessels and LNs (representative dot plots are shown in Fig. 1b). Furthermore, CD2^−^ NK cells were present in AL (*P* < 0·001) and LNs (*P* < 0·001) at a significantly higher proportion compared with PB (Fig. 1d).

### CD2^−^ NK cells are primed in AL and LNs

**Figure 2:**
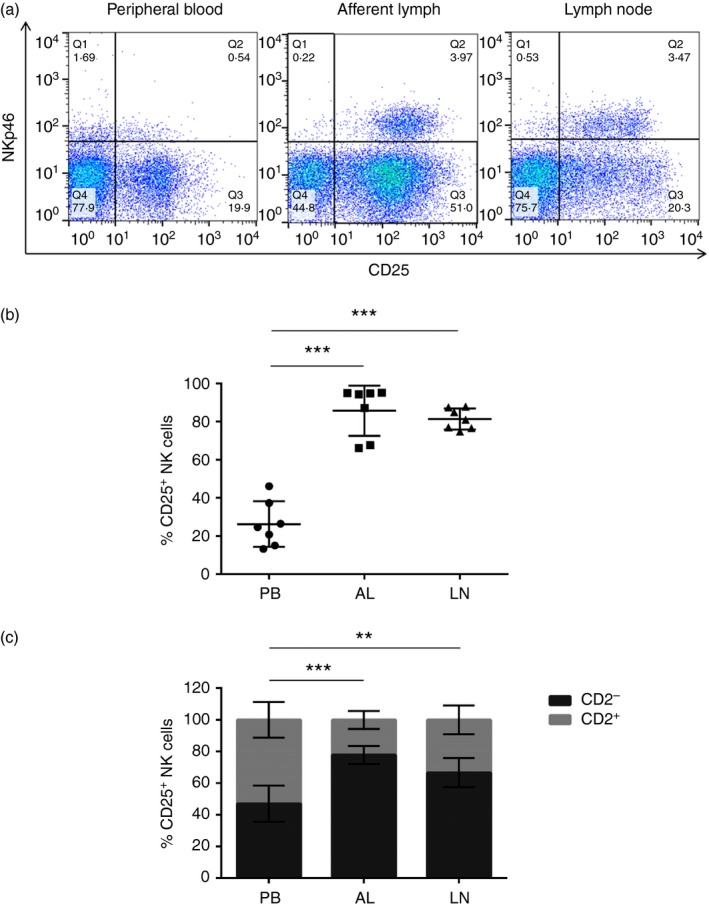
CD2^−^ natural killer (NK) cells are primed in afferent lymph (AL) and lymph nodes (LNs). Lymphocytes from PB, AL and LNs of seven calves were labelled with monoclonal antibodies for NKp46, CD2 and CD25 and analysed by flow cytometry. FACS plots from one representative animal illustrate the expression of NKp46 and CD25 by PB‐, AL‐ and LN‐derived lymphocytes (a). Gates were set based on Fluorescence Minus One controls. Pooled data from seven calves illustrate the average percentage of CD25^+^ NK cells ± SD within the total gated NKp46^+^ NK cell population from PB (circles), AL (squares) or LNs (triangles) (b). Pooled data from seven calves illustrate the average percentage of CD2^+^ (lighter bars) and CD2^−^ (darker bars) NK cells ± SD within the total gated NKp46^+^ CD25^+^ population from PB, AL or LNs (c). Data were normally distributed (*P* > 0·05). Significance between PB and AL was assessed using a paired *t*‐test and significance between PB and LN was assessed using a two‐sample *t*‐test. *P* < 0·01**, *P* < 0·001***. [Colour figure can be viewed at wileyonlinelibrary.com]

The activation status of NK cells derived from PB, AL and the LNs was analysed by determining the expression of the surrogate activation marker CD25 (representative dot plots are shown in Fig. 2a). In contrast to PB, where 26·3% (13·3–46·2%; SD = 10·9) of NK cells were CD25^+^, the percentage of CD25^+^ NK cells was significantly increased in AL (*P* < 0·001) and LNs (*P* < 0·001), with 85·8% (66·1–95·2%; SD = 12·2) and 81·4% (74·8–87·9%; SD = 5·1) of AL‐derived and LN‐derived NK cells expressing CD25 respectively (Fig. 2b). To define the subset of NK cells responsible for the increased activation of NK cells in AL and LNs, the percentage of CD2^+^ and CD2^−^ NK cells within the CD25^+^ population from PB, AL and LNs was assessed. CD25 was equally expressed by PB derived CD2^+^ and CD2^−^ NK cells; however, the percentage of CD2^−^ CD25^+^ NK cells increased significantly in AL (*P* < 0·001) and the LNs (*P* = 0·005; Fig. 2c).

### CD62L expression by AL derived CD2^−^ NK cells may permit homing to LNs

**Figure 3:**
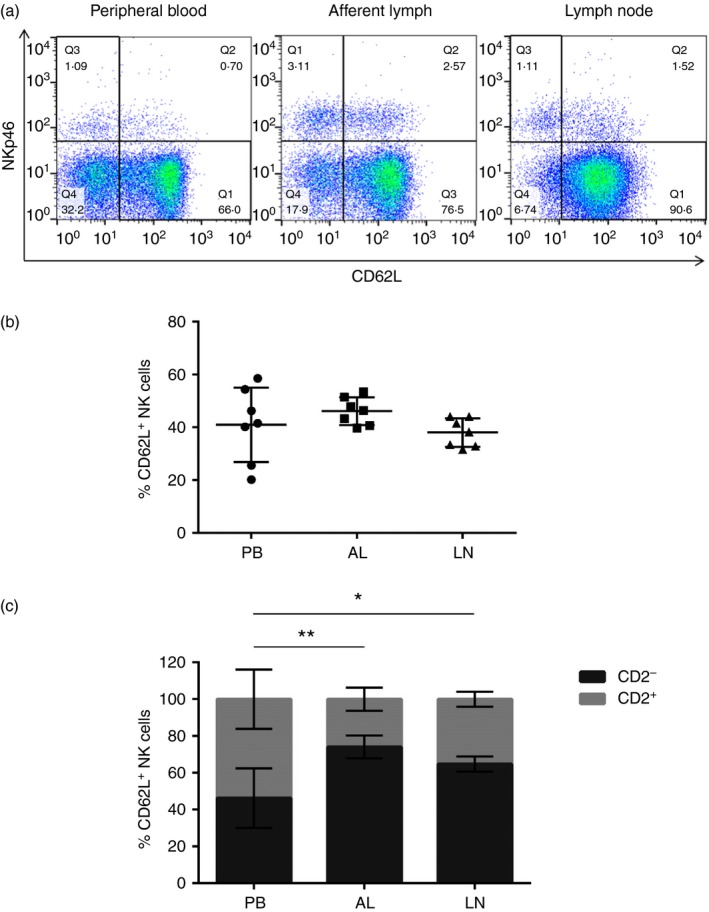
CD62L expression by afferent‐lymph (AL) ‐derived CD2^−^ natural killer (NK) cells may permit homing to lymph nodes (LNs). Lymphocytes from peripheral blood (PB), AL and LNs from seven calves were labelled with monoclonal antibodies for NKp46, CD2 and CD62L and analysed by flow cytometry. FACS plots from one representative animal illustrate the expression of NKp46 and CD62L by PB‐, AL‐ and LN‐derived lymphocytes (a). Gates were set based on Fluorescence Minus One controls. Pooled data from seven calves indicate the average percentage of NKp46^+^ CD62L^+^ NK cells ± SD present across the three compartments (b). Pooled data from seven calves illustrates the average percentage of CD2^+^ (lighter bars) and CD2^−^ (darker bars) NK cells ± SD within the total gated NKp46^+^ CD62L^+^ population from PB, AL or LNs (c). Significance between PB and AL was assessed using a paired *t*‐test and significance between PB and LN was assessed using a two‐sample *t*‐test. *P* < 0·05*, *P* < 0·01**. [Colour figure can be viewed at wileyonlinelibrary.com]

To assess the migratory potential of NK cells from different compartments, the expression of the LN homing molecules CD62L (l‐selectin; representative dot plots are shown in Fig. 3a) and CCR7 by PB‐, AL‐ and LN‐derived NK cells was assessed. No significant differences between the percentages of CD62L^+^ NK cells present across PB, AL and LNs were observed (Fig. 3b). However, the percentage of CD2^***−***^ NK cells expressing CD62L was significantly increased in AL (*P* = 0·004) and LNs (*P* = 0·024), compared with PB (Fig. 3c). CCR7 expression was not detected on PB‐ and LN‐derived NK cells and the expression was highly variable on AL‐derived NK cells (data not shown).

### CD2^***+***^ NK cells are the predominant subset of NK cells present in bovine skin

**Figure 4:**
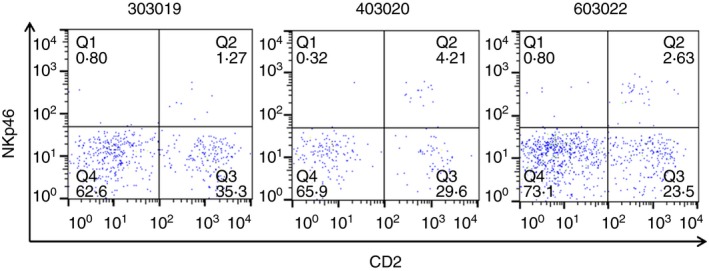
CD2^+^ natural killer (NK) cells are the predominant subset of NK cells present in bovine skin. Pieces of skin were removed from calves at post mortem and enzymatically digested to obtain lymphocytes. Lymphocytes were labelled with monoclonal antibodies to NKp46 and CD2 and analysed by flow cytometry. FACS plots from three calves (303019, 403020 and 603022) illustrate the expression of NKp46 and CD2 by skin‐derived lymphocytes. Quadrants were set based on Fluorescence Minus One controls. [Colour figure can be viewed at wileyonlinelibrary.com]

Data presented so far provide evidence that bovine NK cells are present within skin‐draining afferent lymphatic vessels in steady‐state conditions. However, it is not known if NK cells are resident in the skin of cattle in steady‐state conditions and if their presence in the skin‐draining afferent lymphatic vessels reflects migration from the skin or recruitment from elsewhere. Therefore, pieces of skin were enzymatically digested and assessed for the presence of NK cells. NKp46^+^ NK cells were present in the skin of three calves and accounted for a mean of 2·9% (1·5–4·2%; SD = 1·1) of the lymphocytes present in the skin. CD2^+^ NK cells were the main subset of NK cells present in the skin and accounted for 83·9% (77·3–96·2%; SD = 8·8) of NK cells present (Fig. 4).

### NK cells are present in bovine efferent lymph and CD2^−^ NK cells are the principal subset present

**Figure 5:**
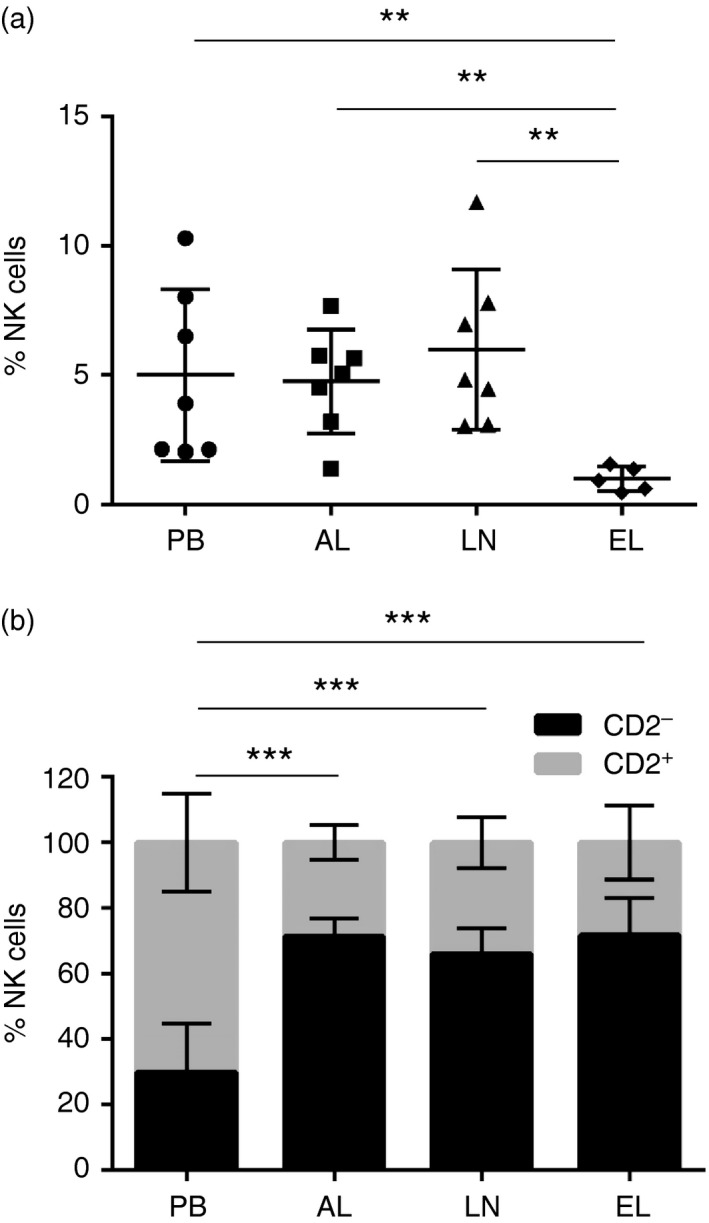
Natural killer (NK) cells are present in bovine efferent lymph and CD2^−^ NK cells are the principal subset present. Lymphocytes derived from peripheral blood (PB), afferent lymph (AL), lymph nodes (LNs) and efferent lymph (EL) were labelled with monoclonal antibodies for NKp46, CD3 and CD2 and analysed by flow cytometry. Pooled data from seven calves for PB, AL and LNs and five calves for EL, show the average percentage of NKp46^+^ CD3^−^ NK cells ± SD present within PB (circles), AL (squares), LNs (triangles) and EL (diamonds) (a). Pooled data from seven (PB, AL and LNs) and five (EL) calves illustrate the average percentage of NKp46^+^ CD2^+^ (lighter bars) and NKp46^+^ CD2^−^ (darker bars) NK cells ± SD within the total gated NKp46^+^ NK cell population from PB, AL, LNs and five calves for EL (b). Data were normally distributed (*P* > 0·05) and significance between PB and EL was assessed using a two‐sample *t*‐test. *P* < 0·01**, *P* < 0·001***.

To address whether bovine NK cells were present in efferent lymph and therefore able to egress from the LNs via the efferent lymphatic vessel in steady‐state conditions, we assessed NK cells in efferent lymph samples of five separate calves and compared these with matched samples of PB. Initially, we determined whether the cellular composition of the PB from the efferent lymphatic cannulated calves was similar to the composition of the PB from the afferent lymphatic cannulated calves (as illustrated in Figs 1, 2, 3). Hence, the frequency and phenotype of NK cells derived from matched PB and EL from three calves was investigated (see Supplementary material, Fig. S2a–d). After confirming the samples of PB from the efferent lymphatic cannulated calves (Fig. S2a–d) were of a similar cellular composition to the PB from the afferent lymphatic cannulated calves (Figs 1, 2, 3), the frequency and phenotype of EL‐derived NK cells were directly compared with NK cells from PB, AL and LNs. NK cells represented 1% (0·48–1·58%; SD = 0·4) of lymphocytes within EL, and NK cell frequency was significantly lower in EL compared with PB (*P* = 0·020), AL (*P* = 0·003) and LNs (*P* = 0·006; Fig. 5a). Similar to NK cells present in AL and LNs, 71·7% (66·8–76·6%; SD = 3·4) of EL‐derived NK cells were CD2^***−***^ and CD2^***−***^ NK cells were present at a significantly higher percentage in EL compared with PB (*P* < 0·001; Fig. 5b).

### CD2^−^ NK cells are primed in efferent lymph

**Figure 6:**
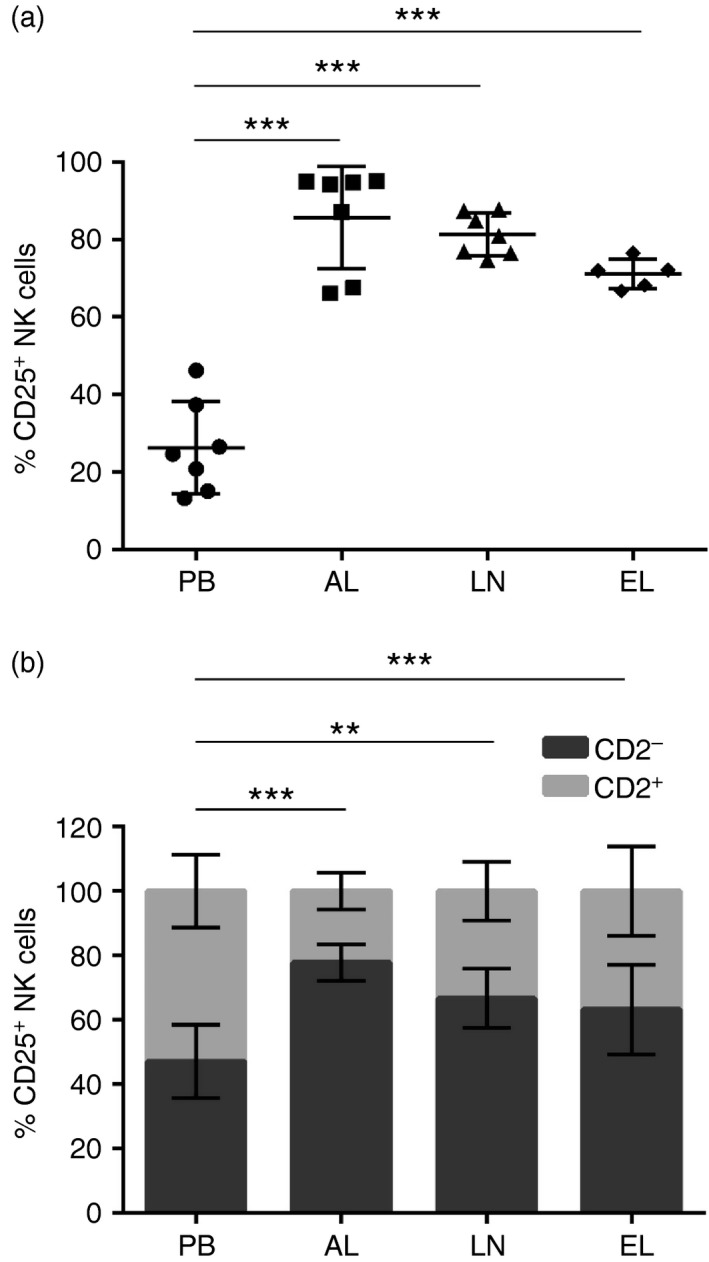
CD2^***−***^ natural killer (NK) cells are primed in efferent lymph. Lymphocytes derived from peripheral blood (PB), afferent lymph (AL), lymph nodes (LNs) and efferent lymph (EL) were labelled with monoclonal antibodies to NKp46, CD2 and CD25 and analysed by flow cytometry. Pooled data from seven calves for PB, AL and LNs, and five calves for EL, illustrate the average percentage of CD25^+^ NK cells ± SD within the total NKp46^+^ population from PB (circles), AL (squares), LNs (triangles) and EL (diamonds) (a). Pooled data from seven (PB, AL and LNs) and five (EL) calves shows the average percentage of NKp46^+^ CD2^+^ (lighter bars) and NKp46^+^ CD2^***−***^ (darker bars) NK cells ± SD within the total gated NKp46^+^ CD25^+^ NK cell population from PB, AL, LN and EL (b). Data were normally distributed (*P* > 0·05) and significance between PB and EL was assessed using a two‐sample *t*‐test. *P* < 0·05*, *P* < 0·01**, *P* < 0·001***.

The expression of CD25 by EL‐derived NK cells was compared with NK cells from PB, AL and LNs. Of the NK cells from EL, 71·2% (66·8–76·6%; SD = 3·4) were CD25^+^ and, similar to those present in AL and LNs, the percentage of CD25^+^ NK cells was significantly increased in EL (*P* < 0·001) compared with PB (Fig. 6a). Furthermore, when assessing the subset of NK cells responsible for this enhanced activation of NK cells in the EL, 63·3% (45·1–77·3%; SD = 12·5) of CD25^+^ NK cells were CD2^***−***^; however, this was not significantly (*P* = 0·069) different to the percentage of CD2^***−***^ CD25^+^ NK cells present in PB, AL or LNs (Fig. 6b).

## Discussion

The experiments described in this manuscript aimed to identify and characterize NK cell phenotypic subsets across different compartments in cattle. It was demonstrated recently that bovine NK cells are present within the afferent lymphatic vessels draining the skin of cattle in the steady‐state, suggesting that they can traffic from tissues into LNs via the lymphatics.^23^ We extended these findings by comparing NK cells from PB, AL and LNs in the same animal, using a total of seven calves. Furthermore, we determined whether NK cells could egress from the LNs via the efferent lymphatic vessel to return to circulation.

NKp46^+^ CD3^−^ NK cells were present in AL draining the skin of calves in the steady‐state in a similar proportion to that found in PB (Fig. 1c), illustrating that bovine NK cells can traffic from tissues such as the skin to the draining LNs via afferent lymphatic vessels. In accordance with these findings, NK cells have been reported to be more abundant in PB than AL in a study involving non‐matched samples of PB and AL.^23^ Furthermore in humans, NK cells were found to be present at significantly lower levels in the AL compared with PB,^16^, ^17^ and recently NK cells were shown to be present in an accumulation of AL representing > 2% of lymphocytes.^18^ In the present study, bovine LNs contained the highest percentage of NK cells (6%; 3·03–11·7%; SD = 2·9) across the three compartments examined (Fig. 1c). This abundance of NK cells within bovine LNs is in accordance with published findings^8^ and is also similar to the increased number of NK cells present in human LNs in steady‐state conditions.^29^ This is in contrast to mouse LNs, where NK cells account for only approximately 0·5% of lymphocytes present.^6^

It has been shown in bovine PB that the majority of NK cells are CD2^+^, whereas CD2^***−***^ NK cells are the predominant subset found within LNs.^8^ Data presented in this study (Fig. 1d) support these findings whereby the percentage of CD2^***−***^ NK cells present within the PB was low, but was significantly increased in AL and LNs, suggesting preferential recruitment of the CD2^***−***^ subset of NK cells from the tissues to the draining LNs in steady‐state conditions. In studies of human afferent lymph, CD56^bright^ NK cells, which are equivalent to bovine CD2^***−***^ NK cells,^30^ were the main subset of NK cells present.^18^

Both AL and LNs draining the skin of cattle in steady‐state conditions contained highly activated NK cells, as defined by cell‐surface expression of CD25, compared with those found in PB (Fig. 2b). CD2^***−***^ NK cells were more activated in AL and LNs than CD2^+^ NK cells (Fig. 2c). In accordance with this finding, bovine CD2^***−***^ NK cells have been shown to be preferentially activated when stimulated with interleukin‐2.^31^ Furthermore, the expression of a second activation marker, CD44, was found to be significantly increased in AL NK cells, which highlights an overall activated phenotype within AL.^23^ This resembles the activated CD4^+^ and CD8^+^ T cells, expressing CD25, CD69 and MHC class II, found in the AL of humans in steady‐state conditions.^16^ Taken together, these findings indicate that events occurring as a result of egress from the skin, result in the arrival of tissue‐activated NK cells in the AL and LNs.

The migratory potential of NK cells derived from PB, AL and LNs was characterized by assessing CD62L and CCR7 expression. Through interactions with high endothelial venules, NK cell expression of CD62L allows homing to LNs.^32^ Whereas there were no significant differences in the percentage of CD62L^+^ NK cells across the three compartments (Fig. 3b), differences in the percentage of CD2^+^ and CD2^***−***^ NK cells expressing CD62L were evident, with an increased percentage of CD2^***−***^ CD62L^+^ NK cells in the AL and LNs, compared with PB (Fig. 3c). This elevated expression of CD62L by CD2^***−***^ NK cells in the AL and LNs correlates with the prevalence of CD2^***−***^ NK cells within these two compartments. Bovine NK cells transcribe the LN homing chemokine receptor CCR7,^30^ but surface CCR7 expression could not be consistently detected by PB‐ or LN‐derived NK cells (data not shown). Using an anti‐human CCR7 antibody cross‐reactive to bovine cells, the expression of CCR7 by AL NK cells was very variable across the seven calves examined (data not shown) and expression was not detected by AL NK cells in previously published work.^23^ In humans, CCR7 is expressed by CD56^bright^ NK cells but is absent from CD56^dim^ NK cells, which coincides with the dominance of the CD56^bright^ subset of NK cells in AL and LNs.^33^ Bovine *γδ* T cells traffic to LNs in a CCR7 independent manner^28^ and therefore bovine NK cells may also move to the LNs in a similar CCR7‐independent manner. Further investigation is required to decipher key molecules used to permit NK cell migration in cattle.

Natural killer cells were found to be resident within bovine skin and the majority were CD2^+^ (Fig. 4). In comparison to CD2^***−***^ NK cells, which transcribe lymphoid homing chemokine receptors, CD2^+^ NK cells are more likely to be non‐migratory due to the absence of these receptors and this may explain their abundance in the skin.^30^ The skin samples analysed in this study were not from the calves used for pseudo‐afferent lymphatic cannulation, and therefore further studies sampling skin from afferent lymphatic cannulated calves would be required to allow direct comparison of skin, PB‐, AL‐ and LN‐derived NK cells in the same animal. Nevertheless, this preliminary experiment provides novel evidence that NK cells are found in the skin of calves and therefore suggests that NK cells can migrate from the skin, through afferent lymphatic vessels and into the LNs. In humans, CD56^bright^ CD16^−^ NK cells are present in the dermis of healthy skin and were also found in skin from patients with atopic eczema/dermatitis, where they were in close contact with CD1a^+^ dendritic cells.^34^, ^35^

After establishing that NK cells can enter draining LNs via afferent lymphatic vessels in steady‐state conditions, samples of EL were analysed for the presence or absence of NK cells to determine if they re‐circulate. NK cells were present in EL, albeit at lower levels than those found in PB, AL and LNs, providing evidence that a proportion of NK cells can exit the LNs through the efferent lymphatic vessel and re‐circulate in steady‐state conditions (Fig. 5a). Similar to NK cells in AL and LNs, EL‐derived NK cells were predominantly CD2^***−***^ (Fig. 5b), indicating that this may be the preferred subset to egress from the nodes and return to the circulation. To date, one study has shown that NK cells are present within human EL, and that these are predominantly of the CD56^bright^ subset;^19^ however, the presence of NK cells in the EL of other species has not been defined. Hence, it appears that in cattle and in humans, CD2^***−***^ NK cells and CD56^bright^ NK cells, which are the principal subsets of NK cells within the LNs, are also the main subsets egressing from the LNs to return to circulation. In conjunction with AL‐ and LN‐derived NK cells, NK cells within EL were activated (Fig. 6a), which was attributed to the CD2^***−***^ subset of NK cells (Fig. 6b). Similarly, human EL‐derived NK cells display an activated phenotype reflected by their increased expression of CD16 and killer cell immunoglobulin‐like receptor in comparison with NK cells in the LNs.^19^ Therefore, it is conceivable that tissue‐activated CD2^***−***^ CD25^+^ NK cells arriving in the LNs also egress from the LNs through the efferent lymphatic vessel and circulate in a primed state in steady‐state conditions. T and B cells use sphingosine‐phosphate‐receptor 1 (S1P1) to egress from peripheral lymphoid organs;^36^, ^37^ however, down‐regulation of S1P1 through administration of FTY720, did not deplete NK cells from the circulation of mice or humans.^38^, ^39^ Consequently, it was demonstrated that NK cells express sphingosine‐phosphate‐receptor 5 (S1P5), which permits NK cell exit from the bone marrow and LNs in mice.^40^, ^41^ It would be valuable to assess the expression of S1P receptors by bovine NK cells to understand the molecules required to exit peripheral lymphoid organs.

In summary, this study has demonstrated that bovine NK cells are present in the skin of cattle in steady‐state conditions and that the CD2^***−***^ subset of NK cells are preferentially recruited, through their expression of CD62L, into draining LNs through afferent lymphatic vessels in a primed state (as defined by the expression of CD25). Similarly, NK cells within bovine LNs are predominantly CD2^***−***^ NK cells with a high expression of CD25. Furthermore, the presence of CD2^***−***^ CD25^+^ NK cells within EL provides novel evidence that a proportion of NK cells do not terminally reside in the LNs and can egress from the nodes to return to circulation where they are predominantly CD2^+^ CD25^lo^. The presence of NK cells within EL is a novel finding in the bovine system with NK cells having only been identified in human EL to date. Therefore we propose that CD2^***−***^ NK cells may represent a recirculating population of lymphocytes in cattle and so may play a role in immune surveillance. Furthermore, previous studies in our laboratory have shown that the CD2^***−***^ subset of bovine NK cells are preferentially activated following *in vitro* co‐culture with Bacillus Calmette–Guérin‐infected dendritic cells^42^ and *Mycobacterium bovis*‐infected dendritic cells.^30^ Therefore, CD2^***−***^ NK cells may migrate from sites of vaccination or infection to draining lymph nodes where they could interact with populations of antigen‐presenting cells and egress via the efferent lymphatic vessel. Given that they migrate from sites of potential pathogen entry or vaccine administration, the circulation of NK cells through AL to LN (and subsequent egress) could also be important in early immune response induction. Detailed examination of NK cell recirculation in the context of vaccination would determine if NK cells could be used as markers of successful vaccination strategies.

## Disclosure

The authors declare no conflict of interest.

## Supporting information


**Figure S1.** Lymphocyte gating strategy.

**Figure S2.** Comparison between peripheral blood (PB) and efferent lymph (EL) ‐derived natural killer (NK) cells.

## References

[1] Kiessling R , Klein E , Pross H , Wigzell H . “Natural” killer cells in the mouse. II. Cytotoxic cells with specificity for mouse Moloney leukemia cells. Characteristics of the killer cell. Eur J Immunol 1975; 5:117–21. 1086218 10.1002/eji.1830050209

[2] Junqueira‐Kipnis AP , Kipnis A , Jamieson A , Juarrero MG , Diefenbach A , Raulet DH *et al* NK cells respond to pulmonary infection with *Mycobacterium tuberculosis*, but play a minimal role in protection. J Immunol 2003; 171:6039–45. 14634116 10.4049/jimmunol.171.11.6039

[3] Cerwenka A , Lanier LL . Natural killer cells, viruses and cancer. Nat Rev Immunol 2001; 1:41–9. 11905813 10.1038/35095564

[4] Lieke T , Graefe SE , Klauenberg U , Fleischer B , Jacobs T . NK cells contribute to the control of *Trypanosoma cruzi* infection by killing free parasites by perforin‐independent mechanisms. Infect Immun 2004; 72:6817–25. 15557602 10.1128/IAI.72.12.6817-6825.2004 PMC529106

[5] Artavanis‐Tsakonas K , Riley EM . Innate immune response to malaria: rapid induction of IFN‐*γ* from human NK cells by live *Plasmodium falciparum*‐infected erythrocytes. J Immunol 2002; 169:2956–63. 12218109 10.4049/jimmunol.169.6.2956

[6] Gregoire C , Chasson L , Luci C , Tomasello E , Geissmann F , Vivier E *et al* The trafficking of natural killer cells. Immunol Rev 2007; 220:169–82. 17979846 10.1111/j.1600-065X.2007.00563.x PMC7165697

[7] Connelley T , Storset AK , Pemberton A , Machugh N , Brown J , Lund H *et al* NKp46 defines ovine cells that have characteristics corresponding to NK cells. Vet Res 2011; 42:37. 21345198 10.1186/1297-9716-42-37 PMC3055825

[8] Boysen P , Gunnes G , Pende D , Valheim M , Storset AK . Natural killer cells in lymph nodes of healthy calves express CD16 and show both cytotoxic and cytokine‐producing properties. Dev Comp Immunol 2008; 32:773–83. 18177938 10.1016/j.dci.2007.11.006

[9] Mair KH , Essler SE , Patzl M , Storset AK , Saalmuller A , Gerner W . NKp46 expression discriminates porcine NK cells with different functional properties. Eur J Immunol 2012; 42:1261–71. 22539298 10.1002/eji.201141989

[10] Tomasello E , Yessaad N , Gregoire E , Hudspeth K , Luci C , Mavilio D *et al* Mapping of NKp46^+^ cells in healthy human lymphoid and non‐lymphoid tissues. Front Immunol 2012; 3:344. 23181063 10.3389/fimmu.2012.00344 PMC3501723

[11] Gowans JL . The recirculation of lymphocytes from blood to lymph in the rat. J Physiol 1959; 146:54–69. 13655215 10.1113/jphysiol.1959.sp006177 PMC1356889

[12] Masopust D , Schenkel JM . The integration of T cell migration, differentiation and function. Nat Rev Immunol 2013; 13:309–20. 23598650 10.1038/nri3442

[13] Carrega P , Ferlazzo G . Natural killer cell distribution and trafficking in human tissues. Front Immunol 2012; 3:347. 23230434 10.3389/fimmu.2012.00347 PMC3515878

[14] Martin‐Fontecha A , Thomsen LL , Brett S , Gerard C , Lipp M , Lanzavecchia A *et al* Induced recruitment of NK cells to lymph nodes provides IFN‐*γ* for T_H_1 priming. Nat Immunol 2004; 5:1260–5. 15531883 10.1038/ni1138

[15] Bajenoff M , Breart B , Huang AY , Qi H , Cazareth J , Braud VM *et al* Natural killer cell behavior in lymph nodes revealed by static and real‐time imaging. J Exp Med 2006; 203:619–31. 16505138 10.1084/jem.20051474 PMC2118232

[16] Yawalkar N , Hunger RE , Pichler WJ , Braathen LR , Brand CU . Human afferent lymph from normal skin contains an increased number of mainly memory/effector CD4^+^ T cells expressing activation, adhesion and co‐stimulatory molecules. Eur J Immunol 2000; 30:491–7. 10671204 10.1002/1521-4141(200002)30:2<491::AID-IMMU491>3.0.CO;2-H

[17] Hunger RE , Yawalkar N , Braathen LR , Brand CU . The HECA‐452 epitope is highly expressed on lymph cells derived from human skin. Br J Dermatol 1999; 141:565–9. 10583071 10.1046/j.1365-2133.1999.03031.x

[18] Carrega P , Bonaccorsi I , Di Carlo E , Morandi B , Paul P , Rizzello V *et al* CD56^bright^perforin^low^ noncytotoxic human NK cells are abundant in both healthy and neoplastic solid tissues and recirculate to secondary lymphoid organs via afferent lymph. J Immunol 2014; 192:3805–15. 24646734 10.4049/jimmunol.1301889

[19] Romagnani C , Juelke K , Falco M , Morandi B , D'Agostino A , Costa R *et al* CD56^bright^CD16^–^ killer Ig‐like receptor – NK cells display longer telomeres and acquire features of CD56^dim^ NK cells upon activation. J Immunol 2007; 178:4947–55. 17404276 10.4049/jimmunol.178.8.4947

[20] Storset AK , Kulberg S , Berg I , Boysen P , Hope JC , Dissen E . NKp46 defines a subset of bovine leukocytes with natural killer cell characteristics. Eur J Immunol 2004; 34:669–76. 14991596 10.1002/eji.200324504

[21] Cooper MA , Fehniger TA , Caligiuri MA . The biology of human natural killer‐cell subsets. Trends Immunol 2001; 22:633–40. 11698225 10.1016/s1471-4906(01)02060-9

[22] Chiossone L , Chaix J , Fuseri N , Roth C , Vivier E , Walzer T . Maturation of mouse NK cells is a 4‐stage developmental program. Blood 2009; 113:5488–96. 19234143 10.1182/blood-2008-10-187179

[23] Lund H , Boysen P , Hope JC , Sjurseth SK , Storset AK . Natural killer cells in afferent lymph express an activated phenotype and readily produce IFN‐*γ* . Front Immunol 2013; 4:395. 24319444 10.3389/fimmu.2013.00395 PMC3837235

[24] Hope JC , Howard CJ , Prentice H , Charleston B . Isolation and purification of afferent lymph dendritic cells that drain the skin of cattle. Nat Protoc 2006; 1:982–7. 17406334 10.1038/nprot.2006.125

[25] Schwartz‐Cornil I , Epardaud M , Bonneau M . Cervical duct cannulation in sheep for collection of afferent lymph dendritic cells from head tissues. Nat Protoc 2006; 1:874–9. 17406320 10.1038/nprot.2006.147

[26] Johnson LA , Jackson DG . Cell traffic and the lymphatic endothelium. Ann N Y Acad Sci 2008; 1131:119–33. 18519965 10.1196/annals.1413.011

[27] Liu L , Zhang M , Jenkins C , MacPherson GG . Dendritic cell heterogeneity *in vivo*: two functionally different dendritic cell populations in rat intestinal lymph can be distinguished by CD4 expression. J Immunol 1998; 161:1146–55. 9686573

[28] Vrieling M , Santema W , Van Rhijn I , Rutten V , Koets A . *γδ* T cell homing to skin and migration to skin‐draining lymph nodes is CCR7 independent. J Immunol 2012; 188:578–84. 22156593 10.4049/jimmunol.1101972

[29] Fehniger TA , Cooper MA , Nuovo GJ , Cella M , Facchetti F , Colonna M *et al* CD56^bright^ natural killer cells are present in human lymph nodes and are activated by T cell‐derived IL‐2: a potential new link between adaptive and innate immunity. Blood 2003; 101:3052–7. 12480696 10.1182/blood-2002-09-2876

[30] Siddiqui N , Hope J . Differential recruitment and activation of natural killer cell sub‐populations by *Mycobacterium bovis*‐infected dendritic cells. Eur J Immunol 2013; 43:159–69. 23124835 10.1002/eji.201242736

[31] Boysen P , Olsen I , Berg I , Kulberg S , Johansen GM , Storset AK . Bovine CD2^–^/NKp46^+^ cells are fully functional natural killer cells with a high activation status. BMC Immunol 2006; 7:10. 16643649 10.1186/1471-2172-7-10 PMC1482717

[32] Chen S , Kawashima H , Lowe JB , Lanier LL , Fukuda M . Suppression of tumor formation in lymph nodes by L‐selectin‐mediated natural killer cell recruitment. J Exp Med 2005; 202:1679–89. 16352740 10.1084/jem.20051473 PMC2212958

[33] Maghazachi AA . Role of chemokines in the biology of natural killer cells. Curr Top Microbiol Immunol 2010; 341:37–58. 20369317 10.1007/82_2010_20

[34] Buentke E , Heffler LC , Wilson JL , Wallin RP , Lofman C , Chambers BJ *et al* Natural killer and dendritic cell contact in lesional atopic dermatitis skin–*Malassezia*‐influenced cell interaction. J Invest Dermatol 2002; 119:850–7. 12406330 10.1046/j.1523-1747.2002.00132.x

[35] Ebert LM , Meuter S , Moser B . Homing and function of human skin *γδ* T cells and NK cells: relevance for tumor surveillance. J Immunol 2006; 176:4331–6. 16547270 10.4049/jimmunol.176.7.4331

[36] Matloubian M , Lo CG , Cinamon G , Lesneski MJ , Xu Y , Brinkmann V *et al* Lymphocyte egress from thymus and peripheral lymphoid organs is dependent on S1P receptor 1. Nature 2004; 427:355–60. 14737169 10.1038/nature02284

[37] Pappu R , Schwab SR , Cornelissen I , Pereira JP , Regard JB , Xu Y *et al* Promotion of lymphocyte egress into blood and lymph by distinct sources of sphingosine‐1‐phosphate. Science 2007; 316:295–8. 17363629 10.1126/science.1139221

[38] Vaessen LM , van Besouw NM , Mol WM , Ijzermans JN , Weimar W . FTY720 treatment of kidney transplant patients: a differential effect on B cells, naive T cells, memory T cells and NK cells. Transpl Immunol 2006; 15:281–8. 16635750 10.1016/j.trim.2006.02.002

[39] Walzer T , Chiossone L , Chaix J , Calver A , Carozzo C , Garrigue‐Antar L *et al* Natural killer cell trafficking *in vivo* requires a dedicated sphingosine 1‐phosphate receptor. Nat Immunol 2007; 8:1337–44. 17965716 10.1038/ni1523

[40] Mayol K , Biajoux V , Marvel J , Balabanian K , Walzer T . Sequential desensitization of CXCR4 and S1P5 controls natural killer cell trafficking. Blood 2011; 118:4863–71. 21911833 10.1182/blood-2011-06-362574

[41] Jenne CN , Enders A , Rivera R , Watson SR , Bankovich AJ , Pereira JP *et al* T‐bet‐dependent S1P5 expression in NK cells promotes egress from lymph nodes and bone marrow. J Exp Med 2009; 206:2469–81. 19808259 10.1084/jem.20090525 PMC2768857

[42] Hamilton C , Mahan S , Entrican G , Hope J . Interactions between Natural Killer cells and Dendritic cells favour T helper1‐type responses to BCG in calves. Vet Res 2016; 47:85. 27530534 10.1186/s13567-016-0367-4 PMC4988014

