# Supplementary figures and images for: Frequency and phenotype of natural killer cells and natural killer cell subsets in bovine lymphoid compartments and blood
Source: Immunology. 2017 Feb 7;151(1):89–97. doi: 10.1111/imm.12708 (PMC5382329; doi:10.1111/imm.12708)

## Slide 1
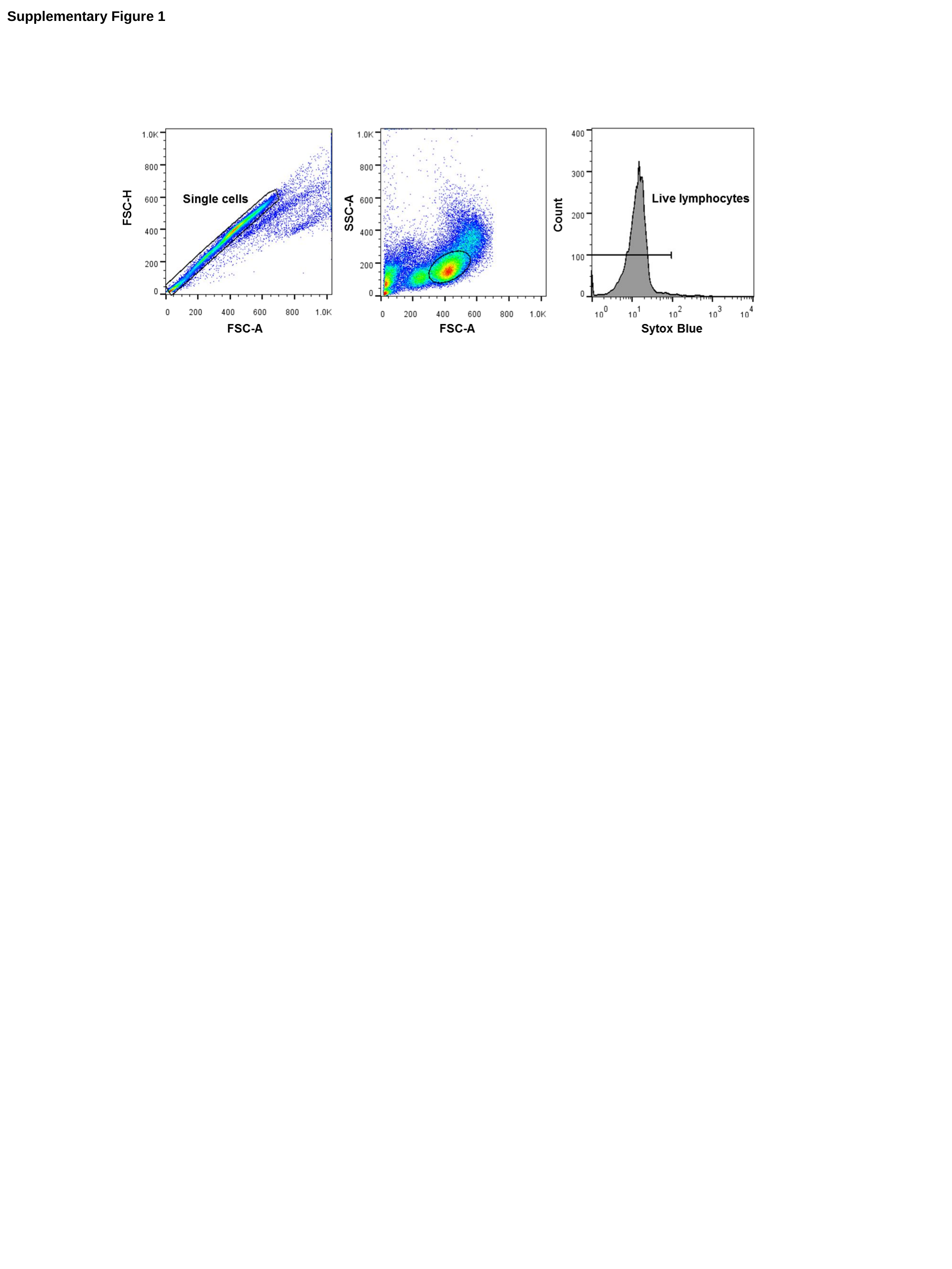

Supplementary Figure 1

Supplement: Supplementary file 1 — Figure S1. Lymphocyte gating strategy. [file IMM-151-89-s001.pptx]

## Slide 1
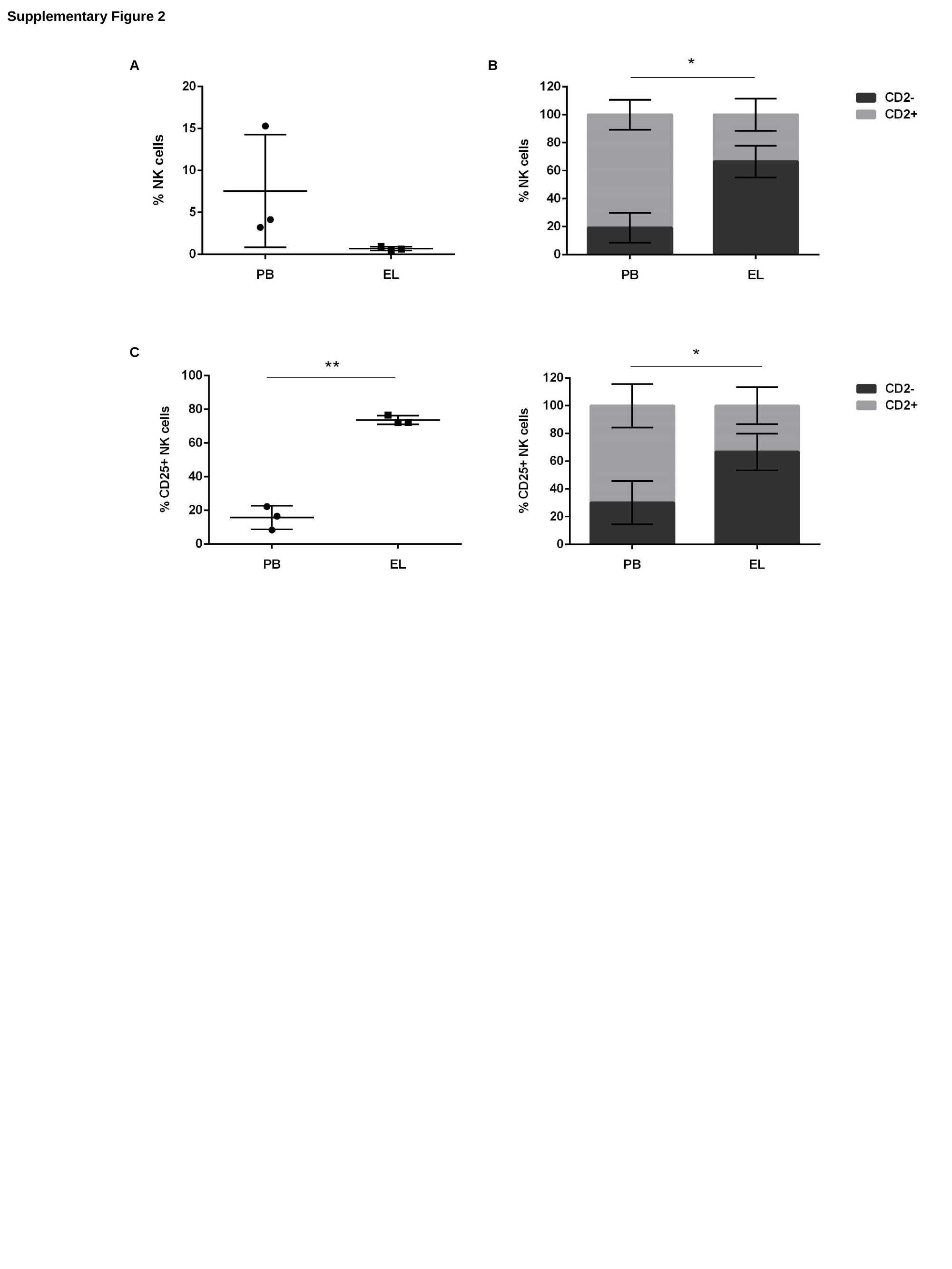

Supplementary Figure 2
B
A
D
C

Supplement: Supplementary file 2 — Figure S2. Comparison between peripheral blood (PB) and efferent lymph (EL) ‐derived natural killer (NK) cells. [file IMM-151-89-s002.pptx]
